# Self-Assembly of Miktoarm Star Polyelectrolytes in Solutions with Various Ionic Strengths

**DOI:** 10.1021/acsomega.2c01317

**Published:** 2022-06-09

**Authors:** Bin Li, Yong-Lei Wang

**Affiliations:** † School of Chemical Engineering and Technology, Sun Yat-sen University, Zhuhai 519082, China; ‡ Department of Materials and Environmental Chemistry, Arrhenius Laboratory, Stockholm University, SE-106 91 Stockholm, Sweden

## Abstract

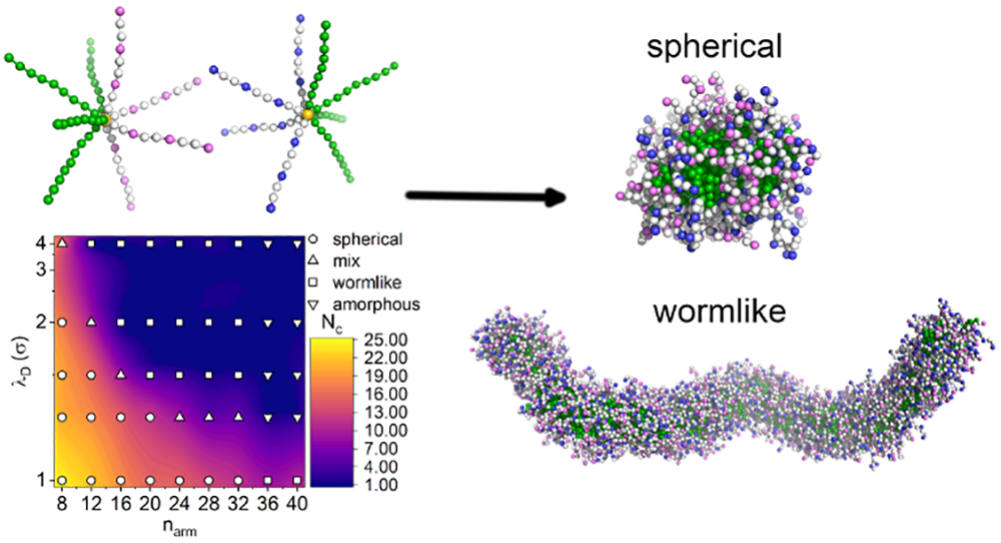

We studied the self-assembly of miktoarm star polyelectrolytes with different numbers of arms in solutions with various ionic strengths using coarse-grained molecular dynamic simulations. Spherical micelles are obtained for star polyelectrolytes with fewer arms, whereas wormlike clusters are obtained for star polyelectrolytes with more arms at a low ionic strength environment, with hydrophilic arms showing a stretched conformation. The number of clusters shows an overall decreasing tendency with increasing the number of arms in star polyelectrolytes due to strong electrostatic coupling between polycations and polyanions. The formation of wormlike clusters follows an overall stepwise pathway with an intermittent association–dissociation process for star polyelectrolytes with weak electrostatic coupling. These computational results can provide relevant physical insights to understand the self-assembly mechanism of star polyelectrolytes in solvents with various ionic strengths and to design star polyelectrolytes with functional groups that can fine-tune self-assembled structures for specific applications.

## 1 Introduction

Soft matter systems have a strong and sensitive response to external perturbations at the room-temperature thermodynamic scale, and thus plenty of nontrivial structures can be obtained via self-assembly of varied building blocks in soft matter systems, such as spherical micelles, wormlike clusters, vesicles, and so on. These self-assembled structures have promising applications in academia and industrial communities, like drug delivery, microreactors, etc.^1−3^ A block copolymer is a representative soft matter system, and the self-assembly of block copolymers has attracted significant attention from researchers during the past decades.^4,5^ Because of significant improvements in synthesis techniques, there are abundant experimental studies in synthesizing block copolymers with complex architectures and varied functional groups. These block copolymers show a striking self-assembly behavior from the corresponding building blocks under varied thermodynamic circumstances and external constraints. The star block copolymers consist of linear polymers (“arms”) attached to a central core, and miktoarm star block copolymers are those having at least two different kinds of arms connected to the central units.^6,7^ The miktoarm star block copolymers can be treated as a “preassembled” cluster of multiple diblock copolymers, and the functionality of miktoarm star block copolymers is strongly correlated with their self-assembly behaviors at varied thermodynamic states.^8−14^ Bačová et al. investigated the influence of arm numbers on self-assembled structures and pathways of poly(ethylene oxide) (PEO)–polystyrene (PS) star polymers.^15,16^ Tsitsilianis and co-workers found that miktoarm star block copolymers tend to self-assemble into micelles with more easily controllable sizes than the corresponding linear block copolymers,^17,18^ which was also observed in our previous simulation study.^19^

The self-assembly of ampliphilic star block copolymers in aqueous solutions is driven by multiple interactions among all the building blocks. Besides hydrophilic and hydrophobic interactions of the building blocks, electrostatic interactions among charged ionic groups have a significant effect on the self-assembly behavior, especially in polymers containing ionic repeating units, so-called polyelectrolytes. A system containing both polycations and polyanions leads to complex polyion coacervation driven by electrostatic associations between oppositely charged repeating units.^20−28^ In addition, electrostatic interactions between charged groups on “macro-ions” in weak polyelectrolytes are dependent on the ionic strength (ion valency and concentration) in solution, according to Debye–Hückel (DH) theory. Thus, the ionic strength and pH values of the solution provide extensive flexibilities to control the self-assembled structures and morphologies of weak polyelectrolytes in solution, which are driven by the electrostatic interactions and release of screened counterions.^20,29−34^

As addressed in previous paragraphs, the self-assembly behavior of miktoarm star block copolymers depends on their architectures, like the number of arms. The star shape results in a locally crowded environment, leading to the arm block copolymers having an extended conformation. If the arms contain ionic repeating units, the crowded environment results in a more extended conformation and higher local charge densities of the star polyelectrolytes due to the local Coulombic repulsions.^35−37^ The conformation of star polyelectrolytes also depends on the pH values and ionic strengths.^38,39^ Erhardt et al. obtained supermicelles via the hydrolysis of PS–PMMA [poly(methyl methacrylate)] star polymers.^40^ Tsukruk and co-workers obtained various self-assembled structures with star polyelectrolytes under different pH values, ionic strengths, and temperatures.^41−45^ Herein, in order to reveal the underlying self-assembly mechanism of star polyelectrolytes in aqueous solutions with varied ionic strengths, we performed extensive coarse-grained simulations to investigate the self-assembly pathways and assembled structures of amphiphilic miktoarm star polyelectrolytes (MSPEs) with different molecular conformations at varied thermodynamic states.

## 2 Results and Discussion

### 2.1 Self-Assembled Structures

**Figure 1:**
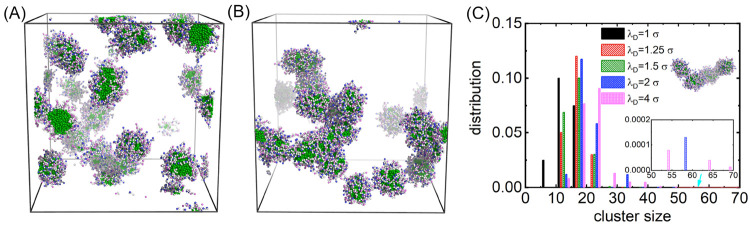
Self-assembled structures formed by MSPEs with the number of arms *n*_arm_ = 8 at (A) λ_D_ = 2.0σ and (B) λ_D_ = 4.0σ. (C) The normalized cluster size distributions for micelles formed by MSPEs with *n*_arm_ = 8 at different ionic strengths, the inset shows the size distribution of wormlike micelles and representative structures.

We built a coarse-grained (CG) model of MSPEs with different arm numbers, the arm length was fixed at nine beads. The MSPE molecules are distributed in aqueous solution in our modeling systems with an implicit solvent and salt model, based on DH theory. The representative molecular architecture is shown in Figure 7, and the detailed information on the model is described in section 4. First of all, we investigated the self-assembly of MSPEs having eight arms (*n*_arm_ = 8), and we found that these MSPEs form spherical micelles if the ionic strength is not too low (from Debye length λ_D_ = 1.0σ to λ_D_ = 2.0σ). Figure 1A shows representative self-assembled structures formed by MSPEs with *n*_arm_ = 8 at λ_D_ = 2.0σ. These micelles show a relatively narrow distribution in cluster sizes, which is similar to our previous findings for self-assembled structures from neutral starlike polymers in aqueous solution.^14^ In addition, some MSPEs form striking aggregates of spherical micelles with a “necklace-like” connection or wormlike micelles with a small aspect ratio at λ_D_ = 4.0σ (Figure 1B), but the majority of self-assembled structures are spherical micelles. The formation of large aggregates at low ionic strength is mainly attributed to the strong electrostatic attractions between hydrophilic arms having oppositely charged beads.

On the basis of these computational results, we calculated the number of MSPEs in micelles (cluster size distributions) formed at different ionic strengths (inverse of Debye lengths). The method for calculating the cluster size is available in the Supporting Information. It is shown in Figure 1C that the cluster size distributions of micelles show striking patterns at different ionic strengths. The narrowest distribution of micelles formed at λ_D_ = 1.0σ corresponds to the micelles having the smallest cluster sizes, which is essentially attributed to the strong screening effect from implicit salts on the charged beads in the MSPEs leading to weak electrostatic coupling between the MSPEs having oppositely charged beads. Such a self-assembly behavior of MSPEs at λ_D_ = 1.0σ (high ionic strength) is akin to the self-assembled structures of the corresponding neutral star block copolymers. A gradual increase in Debye length (decrease in ionic strength) leads to the formation of micelles with large cluster sizes or clusters with “connected” micelles. For example, large clusters consisting of more than 60 MSPEs are formed at λ_D_ = 4.0σ (inset of Figure 1C), which is not observed in the systems at high ionic strengths, indicating that the ionic strength of the modeling systems plays a significant role in tuning the self-assembled structures of the MSPEs in aqueous solution.

**Figure 2:**
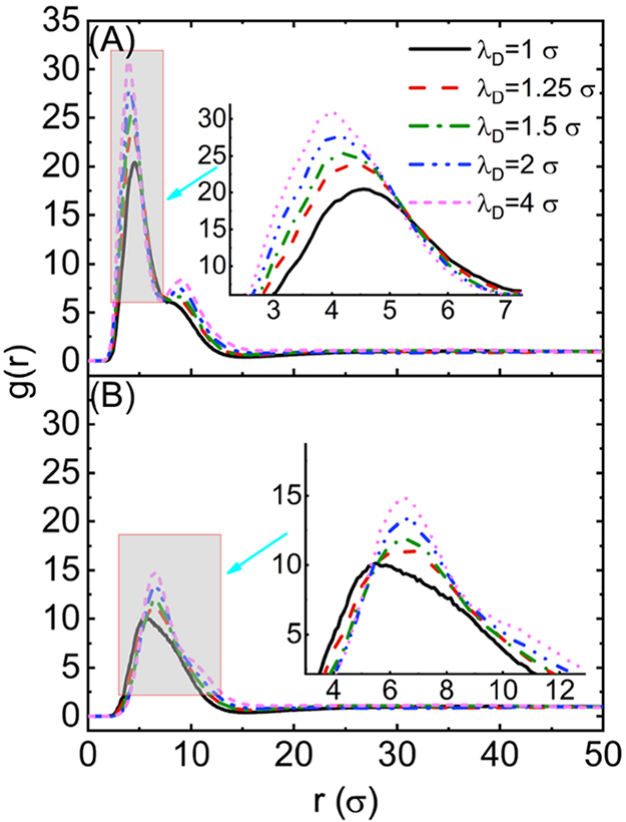
Radial distribution functions between centers of a (A) polycation–polyanion pair and (B) polycation–polycation pair. Peak positions and intensities are clearly manifested in the insets.

Additional analyses of self-assembled structures inside spherical micelles were performed by calculating the radial distribution function (RDF) between the centers of the MSPEs. Computational results of the RDFs between centers of polycation–polyanion and polycation–polycation pairs are illustrated in Figure 2, parts A and B, respectively. The peak positions of the RDFs for centers of oppositely charged MSPEs exhibit a gradual shift to small radial distances with a concomitant increase in Debye length (decrease in ionic strength), indicating that a low ionic strength leads to a strong electrostatic attraction between oppositely charged beads in MSPEs. More pertinently, the strong electrostatic coupling between oppositely charged beads in MSPEs leads to enhanced peak intensities in the RDF plots. This is clearly manifested in the main peaks centered at 4.0σ and in the secondary peaks located around 9.0σ in the RDF plots for self-assembled micelles formed at λ_D_ = 4.0σ compared with those at λ_D_ = 2.0σ, indicating that the centers of the MSPEs in the self-assembled micelles are more significantly aggregated at lower ionic strengths compared with those formed at higher ionic strengths. As expected, the peak positions in the RDF plots between polycations appear at larger radial distances in modeling systems with lower ionic strengths, due to stronger electrostatic repulsions between co-ions (Figure 2B).

**Figure 3:**
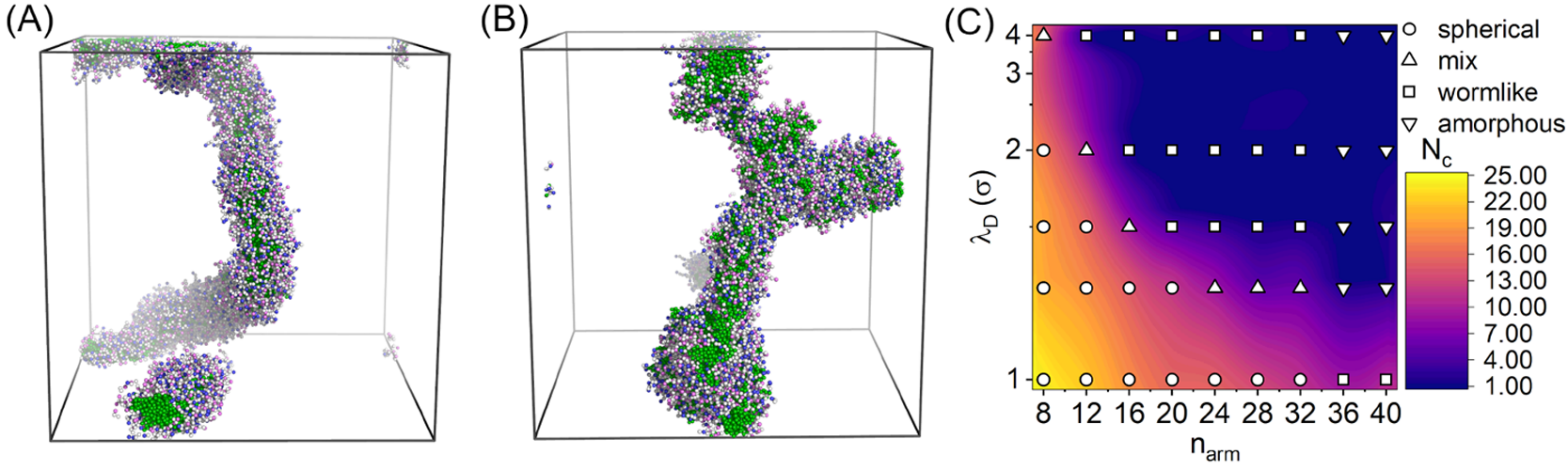
Self-assembled structures of MSPEs at λ_D_ = 2.0σ with (A) *n*_arm_ = 16 and (B) *n*_arm_ = 40. (C) The phase diagram of λ_D_ vs *n*_arm_ of self-assembled structures of MSPEs with explicit numbers (*N*_c_) of formed micelles in the modeling systems.

In the next step, we varied the number of arms in the MSPEs to explore their self-assembled structures in aqueous solution at different ionic strengths. Figure 3A presents a representative wormlike cluster formed by MSPEs with *n*_arm_ = 16 at λ_D_ = 2.0σ, which is distinct from that formed by MSPEs with *n*_arm_ = 8 at the same ionic strength (Figure 1A). The self-assembled structure exhibits a wormlike conformation with a large aspect ratio compared with that formed by MSPEs with *n*_arm_ = 8 at λ_D_ = 4.0σ (Figure 1B). It should be emphasized that MSPEs with *n*_arm_ = 16 still self-assemble into spherical micelles at a strong screening regime, for example, at λ_D_ = 1.0σ (Figure S1). Such an observation indicates that the self-assembled structures of the MSPEs depend on both the number of arms in the MSPEs and the ionic strength of the aqueous solution. An increase in the number of arms in the MSPEs results in a high charge density in the local environment, leading to enhanced electrostatic attractions between the polycations and polyanions, which provides another driving force for clustering of MSPEs. Figure 3B presents representative self-assembled structures of MSPEs with *n*_arm_ = 40 at λ_D_ = 2.0σ, which are characterized by elongated clusters but with some amorphous structures. In addition, the apolar domains formed by the hydrophobic beads in the MSPEs are not fully covered by hydrophilic beads due to the strong condensation effect of oppositely charged beads in the MSPEs.

With systematic changes in the Debye length λ_D_ and the number of arms *n*_arm_, we constructed a phase diagram for the self-assembled structures of MSPEs in aqueous solution with varied ionic strengths, shown in Figure 3C. In order to further differentiate spherical and wormlike micelles, we calculated the radius of gyration tensors of the self-assembled clusters,^14,46,47^ which is defined as
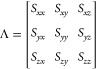
1*S*_*ab*_ = , where *a* and *b* represent the *x*, *y*, or *z* components, respectively. *a*_COM_ and *b*_COM_ are the centers of mass (COM) of the clusters, and *n* is the number of CG beads in the cluster. Diagonalization of matrix Λ gives three eigenvalues, λ_1_, λ_2_, and λ_3_, and herein we set λ_1_ > λ_2_ > λ_3_. The largest eigenvalue λ_1_ represents the radius of gyration along the major axis of the cluster. The asphericity parameter δ was defined to distinguish spherical or wormlike micelles from the three eigenvalues with
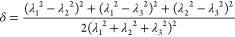
2δ = 0 for a perfect sphere, and δ = 1 for a rod. Herein we define the micelle to be wormlike if δ > 0.3, otherwise, the self-assembled structure is a spherical micelle. Amorphous clusters are determined to be wormlike micelles having multiple “branches”. In addition, the number of formed clusters, *N*_c_, in the modeling systems was determined by averaging simulation trajectories of the last 0.5 × 10^5^τ and are shown as contour plots in Figure 3C. The method for counting the cluster number *N*_c_ is shown in the Supporting Information. A decrease in the ionic strength in aqueous solution leads to the formation of fewer clusters with relatively large cluster sizes. For instance, MSPEs with *n*_arm_ between 12 and 32 assemble into a few wormlike clusters at low ionic strength, whereas smaller spherical micelles are formed if λ_D_ = 1.0σ. For MSPEs with a specific number of arms, the number of formed clusters shows an overall decreasing tendency with the reduction of ionic strength. The self-assembled structures around the border between spherical micelles and wormlike clusters (triangle symbol, purple and pink colors in contour plots in Figure 3C) are mixtures consisting of small wormlike clusters and spherical micelles accompanying the association and dissociation of the wormlike clusters. In most cases, only one or two large wormlike clusters (e.g., Figure 3A) are formed without small spherical micelles (dark blue color in the contour plots in Figure 3C). In modeling systems with MSPEs having an extremely large number of arms (such as *n*_arm_ = 36 or 40), amorphous clusters are observed (Figure 3B) if the screening effect on the polyions is not very strong. The major component of the amorphous clusters is still the elongated structure, but the fusion between different wormlike clusters occurs with relatively random orientations.

**Figure 4:**
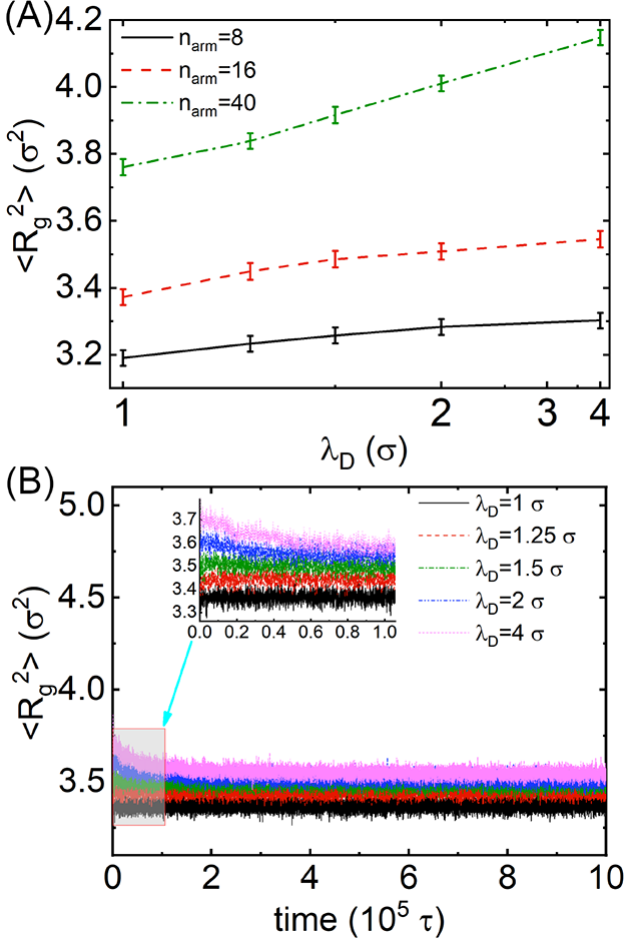
(A) Mean-squared radii of gyration ⟨*R*_g_^2^⟩ of the hydrophilic arms in MSPEs at different ionic strengths. (B) Time evolution of ⟨*R*_g_^2^⟩ of the hydrophilic arms in MSPEs with *n*_arm_ = 16 at different ionic strengths. The inset shows the computational results of the first 1.0 × 10^5^τ.

The radius of gyration values ⟨*R*_g_^2^⟩ of hydrophilic arms in the MSPEs were calculated to further characterize the self-assembled structures of the MSPEs at varied ionic strengths. The values of ⟨*R*_g_^2^⟩ of hydrophilic arms in MSPEs at various ionic strength conditions obtained from the last 0.5 × 10^5^τ are shown in Figure 4A for representative MSPEs with *n*_arm_ = 8, 16, and 40. The full time evolutions of ⟨*R*_g_^2^⟩ for modeling systems consisting of MSPEs with *n*_arm_ = 16 at different ionic strengths are shown in Figure 4B. Hydrophilic arms in MSPEs are more extended in aqueous solution with lower ionic strengths due to strong intramolecular electrostatic repulsions, as expected. The dependence of ⟨*R*_g_^2^⟩ on the number of arms in the MSPEs is more significant than that on ionic strengths in the modeling systems, which is mainly attributed to the fact that MSPEs with more arms have a stronger excluded volume effect and a higher local charge density. However, although hydrophilic arms in MSPEs at lower ionic strength (λ_D_ = 4.0σ) show a decreasing tendency due to strong coupling between polycations and polyanions, the equilibrium ⟨*R*_g_^2^⟩ values are still larger than those at higher ionic strength. The radius of gyration of the hydrophilic arms in MSPEs increases with decreasing ionic strength in solution, as shown in Figure 4B, owing to the stretched conformation of the hydrophilic arms in the modeling systems. Additional computational results of the evolution of ⟨*R*_g_^2^⟩ for MSPEs with *n*_arm_ = 8 and 40 show a similar tendency and are shown in Figure S2.

The values of ⟨*R*_g_^2^⟩ of the hydrophilic arms in MSPEs exhibit a monotonic increase with decreasing ionic strength (Figure 4A), suggesting that the hydrophilic arms in the MSPEs have an extended conformation in the weak screening regime. The more extended hydrophilic blocks often result in the tendency to form spherical micelles rather than wormlike clusters, as the apolar domains are covered by the extending hydrophilic part more completely, if the thermodynamic condition is kept unchanged for the hydrophobic part. However, the phase diagram shown in Figure 3C indicates that MSPEs with identical numbers of arms tend to self-assemble into wormlike clusters at low ionic strengths. It seems that the ⟨*R*_g_^2^⟩ results are not consistent with our assumption above.

**Figure 5:**
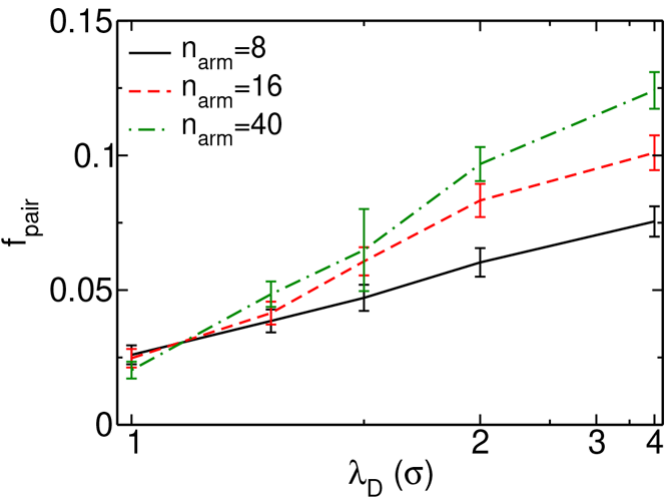
Fraction of paired charged beads in MSPEs with *n*_arm_ = 8, 16, and 40 at different ionic strengths.

In spherical micelles and wormlike clusters, polycations and polyanions are strongly coupled in the low ionic strength regime, resulting in significant overlap of the hydrophilic blocks in the MSPEs due to the electrostatic pairing of oppositely charged beads. In the current work, an ion pair is defined if a positively charged bead can find at least one negatively charged bead within the distance where the RDFs between the positively and negatively charged beads reach the first peak (∼1.15σ as shown in the representative RDF plots in Figure S3). The fraction of positively charged beads paired with negatively charged beads was calculated via *f*_pair_ = *n*_pair_/*n*_pos_, in which *n*_pair_ is the number of formed ion pairs and *n*_pos_ is the total number of positively charged beads in the modeling systems. Figure 5 presents computational results of *f*_pair_ for MSPEs at various ionic strengths with *n*_arm_ = 8, 16, and 40. For a given MSPE, a large proportion of ions will form ion pairs with oppositely charged beads at low ionic strength (large Debye length). In addition, the deviation among *f*_pair_ values in MSPEs with different *n*_arm_’s increases with decreasing ionic strength. In the strong screening regime, for example, λ_D_ = 1.0σ, there is no obvious dependency between *f*_pair_ and *n*_arm_ as both polycations and polyanions exhibit structures similar to those of the corresponding neutral star polymers. On the other hand, MSPEs with more arms show a stronger overlapping effect toward those having oppositely charged beads at the weak screening regime, such as at λ_D_ = 4.0σ, which promotes the formation of wormlike clusters.

### 2.2 Self-Assembly Pathways

**Figure 6:**
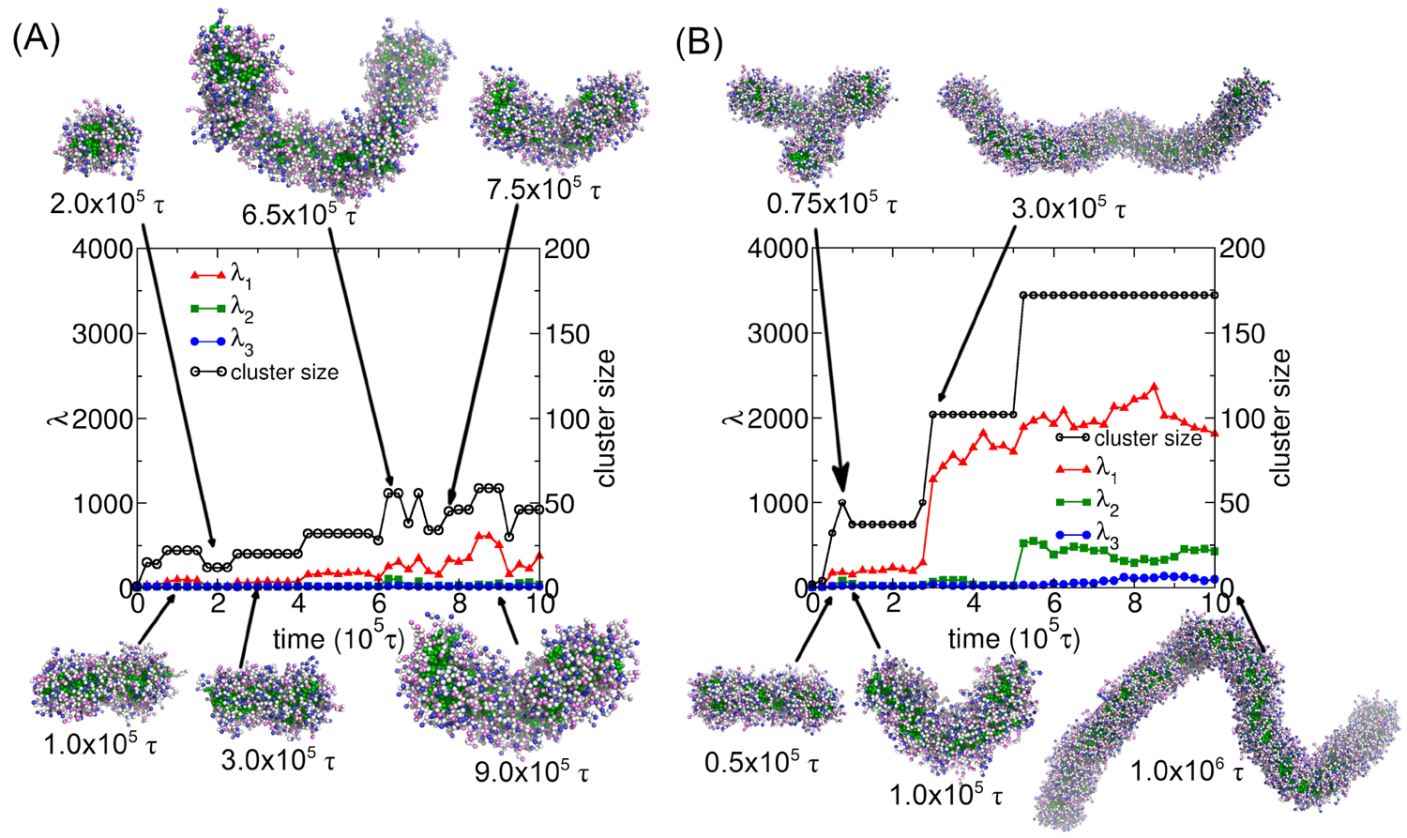
Time evolution of three eigenvalues of the radius of gyration tensors and cluster sizes for the growth of the largest self-assembled wormlike clusters formed by MSPEs with *n*_arm_ = 16 at (A) λ_D_ = 1.5σ and (B) λ_D_ = 2.0σ. Representative snapshots are taken at specific times indicated by the arrows.

Having reconciled the self-assembled structures of the MSPEs in aqueous solution with varied ionic strengths and the number of arms in the MSPEs, we seek to examine the self-assembly pathways for the formation of wormlike clusters. We tracked the growth of the largest self-assembled cluster and the evolution of three eigenvalues λ_1_, λ_2_, and λ_3_ for this cluster in the modeling systems. If the self-assembled structures include several relatively small clusters with either wormlike or spherical shapes, for instance, the structures formed by MSPEs with *n*_arm_ = 16 at λ_D_ = 1.5σ, the self-assembly pathway shows an overall stepwise mechanism, as shown in Figure 6A, even cluster dissociation occurs occasionally. The MSPEs form small wormlike clusters at 1.0 × 10^5^τ, and the eigenvalue of the radius of gyration tensor λ_1_ is slightly larger than that of λ_2_ and λ_3_, corresponding to the formed structures having a wormlike or cylindrical shape. At 2.0 × 10^5^τ, the tracked cluster dissociates from a wormlike to a spherical cluster with three eigenvalues λ_1_ ≈ λ_2_ ≈ λ_3_. As time evolves, this spherical cluster merges with others into a wormlike cluster again at 3.0 × 10^5^τ, but with a smaller cluster size than that formed at 1.0 × 10^5^τ. This wormlike cluster continues to merge with others and forms a larger cluster, the self-assembled wormlike cluster at 6.5 × 10^5^τ exhibits obvious curvature, reflected by a larger λ_2_ value, and cluster dissociation occurs again afterward, leading to the formation of multiple small clusters. The dissociation and association process occurs from 7.0 × 10^5^τ to 9.0 × 10^5^τ again, with representative clusters at 7.5 × 10^5^τ and 9.0 × 10^5^τ shown in Figure 6A. Therefore, the overall self-assembly pathway follows a stepwise increase in cluster sizes and at least one eigenvalue of the radius of gyration tensor, with distinct and intermittent cluster dissociation during the whole process of the simulations.

The MSPEs with *n*_arm_ = 16 also self-assemble into wormlike clusters at λ_D_ = 2.0σ, but the sizes of the wormlike clusters are much larger than that formed at λ_D_ = 1.5σ, resulting in a single cluster in the simulation box (Figure 3C). The self-assembly pathway for this modeling system shows a typical stepwise mechanism with the cluster dissociation occurring rarely, as shown in Figure 6B. A small wormlike cluster is formed at 0.5 × 10^5^τ, the small cluster is associated with another sphere from the side at 0.75 × 10^5^τ, from the observation of the corresponding snapshot shown in Figure 6B and the relatively larger eigenvalue λ_2_, but then the sphere dissociates from the wormlike cluster rapidly at 1.0 × 10^5^τ. The number of MSPEs consisting of this wormlike cluster continues to increase, and they form a larger wormlike cluster at 3.0 × 10^5^τ. The representative snapshot and the corresponding three eigenvalues λ_1_ ≫ λ_2_ ≈ λ_3_ indicate that this cluster is wormlike with a straight conformation and large aspect ratio. This wormlike cluster reaches a size consisting of 102 MSPEs. The elongated cluster merges with other MSPEs after 5.0 × 10^5^τ, resulting in a single large cluster in the system. Although the merging between different clusters occurs as a typical stepwise pathway, the association and dissociation processes were also observed during the clustering. Figure S4 shows that the number of clusters, *N*_c_, oscillates during the clustering process at the time scales of 2.75 × 10^5^τ to 3.0 × 10^5^τ and 5.0 × 10^5^τ to 5.25 × 10^5^τ. It means that some intermediate metastable states appear when the different clusters merge. The cluster shows a relatively large curvature, from the snapshot at 1.0 × 10^6^τ in Figure 6B, as well as larger λ_2_ values. Probably, the large curvature of the cluster is due to the finite size effect, and it is also possible to generate the wormlike cluster including more MSPE molecules in a larger system, but it is beyond our simulation length and time scales.

When the wormlike cluster formed at λ_D_ = 1.5σ (Figure 6A) is compared with that formed at λ_D_ = 2.0σ (Figure 6B), a significant difference is that in the latter case the formation of the wormlike cluster occurs earlier and it grows rapidly to reach a plateau consisting of many MSPEs, whereas in the former case there is the occurrence of a distinct and intermittent association–dissociation process during simulations. Such a phenomenon was also observed for the same MSPEs by observing the cluster number evolutions at different ionic strengths (Figure S5). This is attributed to effective Coulombic attractions between polycations and polyanions, which are stronger at lower ionic strengths leading to a faster fusion between MSPEs carrying oppositely charged beads. If the electrostatic association between the MSPEs is strong enough (low ionic strength), it will provide another driving force together with hydrophobic associations among the hydrophobic arms in the MSPEs to promote the self-assembly of the MSPEs. However, if the electrostatic association between the MSPEs is not so strong (high ionic strength), the hydrophobic association among the hydrophobic arms is the main driving force for the MSPEs to form wormlike clusters, with an intermittent association–dissociation process due to the incompatibility between the hydrophilic and hydrophobic arms in the MSPEs and an overcrowding effect of the hydrophilic corona in the wormlike cluster, or only spherical micelles were formed in simulations. The time evolution of the interaction energies between different components for the two systems is shown in Figure S6, which shows that the hydrophobic and Coulombic interactions drive the self-assembly process collectively (especially at λ_D_ = 2σ), by overcoming the increase of the hydrophilic interactions and the interactions between the two kinds of arms, and converge after long time simulations. In addition, the polyelectrolyte association process is not only driven by the electrostatic attraction between oppositely charged repeat units but also the entropic contribution due to the release of counterions as well as water molecules. The counterions are adsorbed on the ionic repeat units in isolated MSPE molecules, and the ions are covered by solvation shells of water molecules, some other water molecules are also formed as the hydration shell of the polyelectrolytes on the charged groups driven by multiple interactions, for example, hydrogen bonds are formed in sodium poly(styrene-*co*-styrenesulfonate) aqueous solution.^48−50^ Some of the counterions with the solvation shell could be released during the association of oppositely charged MSPEs, due to the excluded volume effect, as well as the dehydration occurs since the hydrogen bonds between the polyelectrolyte and water break. The rest of the “unpaired” charged repeating units are still screened by the counterions in the hydrophilic shell of the aggregates, the different number of counterions that remain in the aggregates, which might be related to the salt concentration, could lead to different self-assembly morphologies, due to the varied screening effect. Our results are reasonable if the counterion and hydration effects are considered. However, the analysis is qualitative because our modeling system adopted an implicit solvent model with DH approximation. Perhaps we could investigate the effect using a model with higher resolution in future work.

## 3 Conclusions

In summary, we have studied the self-assembled structures and pathways of amphiphilic MSPEs at different ionic strengths using coarse-grained molecular dynamics (CGMD) simulations. The self-assembled structures of MSPEs depend on both the number of arms in the MSPEs and the ionic strength in solution. Spherical micelles are obtained by MSPEs with fewer arms, and micelles exhibit distinct structural aggregations at low ionic strength due to the strong electrostatic interactions between the MSPEs. Wormlike clusters are formed by MSPEs with intermediate numbers of arms (less than 36 arms) at low ionic strength, in which hydrophilic arms are less stretched during the self-assembly process of the MSPEs. These wormlike clusters exhibit extended conformation with a gradual increase in ionic strength. The formation of wormlike clusters follows an overall stepwise pathway, and an intermittent association–dissociation process occurs in simulation systems if polycations and polyanions have weak electrostatic coupling. It is expected that these modeling results can provide relevant physical insights for the prediction of self-assembled structures of MSPEs at varied ionic strengths and the design of specific MSPE structures to self-assemble into striking aggregates for promising applications.

## 4 Models and Methods

### 4.1 Coarse-Grained Model

**Figure 7:**
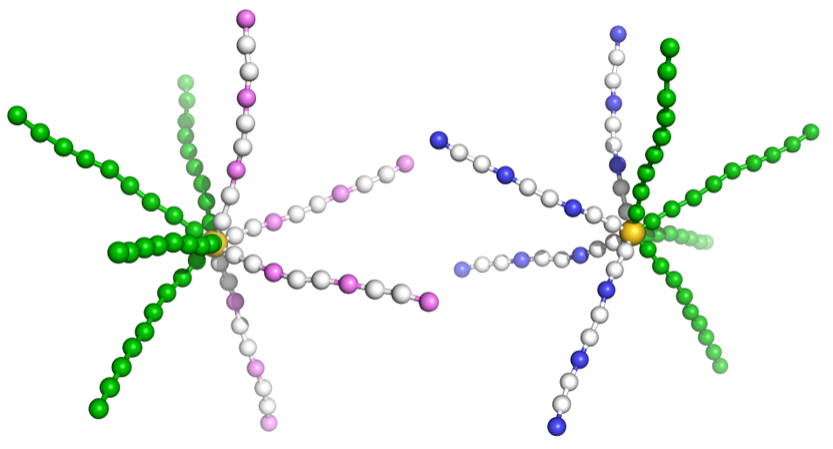
Architecture of a pair of MSPEs, with a polycation and a polyanion. In the two MSPEs, the gold beads represent central cores, violet and blue beads represent positively and negatively charged groups, and white and green spheres correspond to neutral beads with hydrophilic and hydrophobic characters, respectively.

We built the CG model of the MSPEs including one type having positive charges on the arms (“polycation”) and the other type with negative charges on the arms (“polyanion”). Each MSPE has a central core and two kinds of arms, including hydrophobic arms and hydrophilic arms with charged beads. Herein we kept the length of all arms at nine beads and varied the number of arms with respect to an equal arm number for the two types of arms in a single MSPE molecule. The model of a polycation and a polyanion is shown in Figure 7, with every third bead carrying a unit charge. Such a CG model for MSPEs, as addressed in previous publications, can properly describe experimental phase behaviors and self-assembly of star polyelectrolytes in aqueous solution.^51−55^

All CG beads interact with each other via the Lennard-Jones (LJ) potential
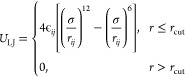
3where ϵ_*ij*_ is the LJ interaction strength between beads *i* and *j*. σ reflects the bead diameter, and we set it to 1.0 for the length unit. The LJ potential is truncated and shifted at the cutoff radius *r*_cut_, beyond which the potential is 0. We set ϵ to 1.0 for LJ interactions between hydrophobic beads and 0.5 between hydrophilic and hydrophobic beads, reflecting the incompatibility between these two kinds of arms.^13,14,56^ The cutoff radius *r*_cut_ is 2.5σ for the aforementioned nonbonded interactions. The interaction strength ϵ = 1.0 between hydrophilic beads is truncated at 2^1/6^σ, which results in a purely repulsive potential.^57^

For hydrophilic arms in two MSPEs, every third bead from the central unit is charged with partial charges of ±1.0*e*. Electrostatic interactions between charged CG beads were based on DH theory, which was developed based on Poisson–Boltzmann (PB) theory, and it provides a feasible route to obtain the analytic solution of the electrostatics potential via a linearized approximation. The application of DH theory on polyelectrolytes has been adopted by many researchers. For instance, the relationship between chain structures and ionic strengths shows the same tendency as the system with explicit salts,^58,59^ which means that the adoption of DH theory reflects the properties of polyelectrolytes qualitatively in some circumstances. The potential was described by a screened Coulombic form including an additional exponential damping factor to the regular Coulombic term
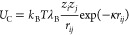
4where *z*_*i*_ and *z*_*j*_ are the charge valencies of beads *i* and *j*. The Bjerrum length, λ_B_ = *e*^2^/(ϵ_r_*k*_B_*T*), defines the length at which the electrostatic interaction energy is equal to the thermal energy scale *k*_B_*T*, with ϵ_r_ being the relative dielectric constant of the solvent, the value of λ_B_ is 7.1 Å for water at ambient condition. Herein we fixed λ_B_ = 3.0σ to mimic the self-assembly of realistic polyelectrolytes, such as sodium poly(styrene-*co*-styrenesulfonate) and poly(acrylamide-*co*-sodium-2-acrylamido-2-methylpropane-sulfonate) in aqueous solution.^52−55,60^ The parameter κ is the inverse of the Debye length, being described as
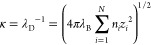
5indicating that the Debye length decreases with an increase in ionic strength. In the current work, the ionic strengths in the modeling systems were tuned by setting different λ_D_ values from λ_D_ = 1σ to 4σ, falling in the range that the chain conformation is close to that in explicit salts with full Coulombic interactions,^58^ and the screening effect of salts on charged beads was reflected by the Debye length implicitly. The cutoff radius of the screened Coulombic potential (eq 4) is set to 5.3λ_D_,^61^ indicating that a system of relatively low ionic strength with a weak electrostatic screening effect experiences longer range interactions. It should be addressed that the adoption of the CG model of star polyelectrolytes with implicit solvent and salt models makes CGMD simulations efficient for studying the self-assembled structures and pathways of star polyelectrolytes in aqueous solution, which, however, might result in the lack of an association–dissociation process of the screening ions on the charged CG beads of the star polyelectrolytes. Nevertheless, the adopted models can provide relevant physical insights of self-assembled structures and morphologies of star polyelectrolytes in aqueous solution with tunable ionic strengths.

The adjacent CG beads are connected via the finite extension nonlinear elastic (FENE) bond potential
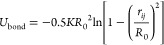
6where *K* denotes the spring constant and is set to 7.0ϵ/σ^2^, *R*_0_ represents the maximum allowed distance between two bonded CG beads and is set to 2.0σ in the current work.^51−55^

### 4.2 Simulation Details

The simulation box length is set to 100σ for all modeling systems, with periodic boundary conditions (PBC) applied in three dimensions. All the simulations are performed under constant bead concentration, each simulation box contains roughly 25 000 CG beads, with equal numbers of polycations and polyanions to keep the system electroneutral, and we assumed that our modeling system corresponds to the condition that the solution pH is away from the p*K*_a_ of each MSPE molecule and they can be close to fully charged.^62^ The equation of motion is updated by following Langevin equation:
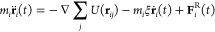
7where ξ is the friction coefficient and is fixed at 1.0τ^–1^, in which τ =  is the time unit. **F**_*i*_^R^ is the random force and satisfies the fluctuation–dissipation theorem:
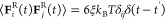
8The coupling of friction and random forces serves as an effective thermostat to mimic solvent implicitly. The simulation temperature is set to 1.2ϵ/*k*_B_ for all modeling systems. All CG MD simulations were performed using the LAMMPS package.^63^ The integration time step was set to δ*t* = 0.005τ, and all CG MD simulations ran 2.0 × 10^8^ steps, to guarantee that the total energies of the modeling systems were converged.

## Supporting Information Available

Method for calculating the cluster size and number of clusters, additional figures, and the validation of the stability of the self-assembled structures (PDF)
