# Supplementary material for: Self-Assembly of Miktoarm Star Polyelectrolytes in Solutions with Various Ionic Strengths
Source: ACS Omega. 2022 Jun 9;7(24):20791–9. doi: 10.1021/acsomega.2c01317 (PMC9219065; doi:10.1021/acsomega.2c01317)
Supplement: Supplementary file 1 — ao2c01317_si_001.pdf [file ao2c01317_si_001.pdf]

Supporting Information for: Self-Assembly of Miktoarm Star-Polyelectrolytes in Solution with Various Ionic Strengths

Bin Li,*[1] and Yong-Lei Wang,*[2]

[1] School of Chemical Engineering and Technology,

Sun Yat-sen University, Zhuhai 519082, China.

E-mail: libin76@mail.sysu.edu.cn

[2] Department of Materials and Environmental Chemistry, Arrhenius Laboratory,

Stockholm University, SE-106 91 Stockholm, Sweden.

E-mail: yonglei.wang@mmk.su.se

Cluster size and number of clusters

First of all, we divided the simulation box into a plenty of small cubic cells, the size of each cell is $1\sigma^3$. Then we assigned the polymer beads on the corresponding cells, based on their coordinates. The first hydrophobic bead that we found by following the sequence of cells was set to the outset of a cluster. Afterwards, we searched for the hydrophobic beads through the neighbor cells of the first hydrophobic bead, if they existed in neighbors, then we continued to search for the hydrophobic beads through the neighbor cells of the new hydrophobic beads we just found. The searching process would not stop until there were no hydrophobic beads in the adjacent cells. All the hydrophobic beads we found belonged to a cluster, and the MSPE molecules including these hydrophobic beads are distributed as a cluster. The above approach was repeated for counting other clusters in the system. Finally, the number of clusters (the parameter N_c in the main article) and cluster sizes were obtained. The heat map for N_c values in Figure 3C were plotted by setting 8 major levels ($N_c = 1$ to 25, as shown in Figure 3C), each major level contains 8 minor levels, to estimate the intermediate values among the simulation system settings (white symbols in Figure 3C). In addition, smoothing is adopted in order to make the heat map to be viewed friendly. We worked on only hydrophobic beads in order to avoid the chain crossing between hydrophilic coronas belong to different clusters.

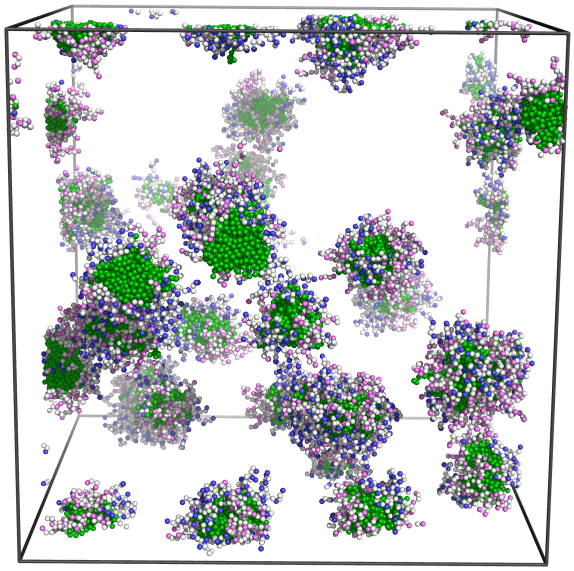

Figure S1: Self-assembly structure formed by MSPEs with $n_{arm} = 16$ under $\lambda_D = 1\sigma$.

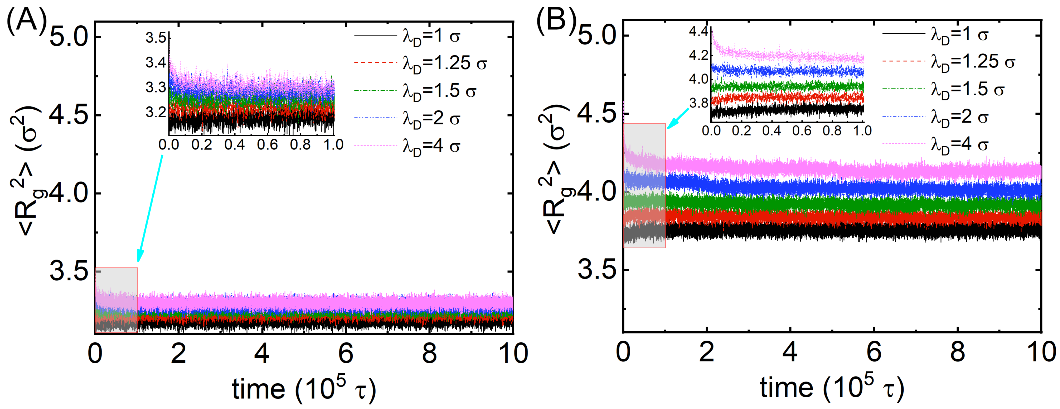

Figure S2: Time evolution of $\langle R_g^2 \rangle$ of hydrophilic arms in MSPEs with $n_{arm} = 8$ (A) and $n_{arm} = 40$ (B), under different ionic strengths (shown in legends). The insets show the results of the first $1.0 \times 10^5 \tau$.

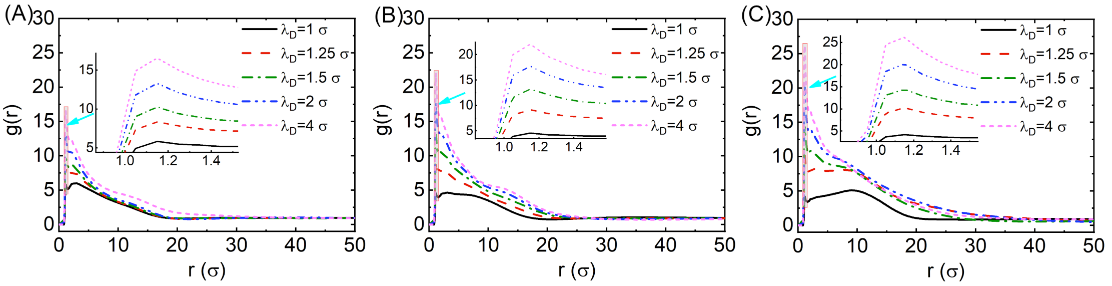

Figure S3: Radial distribution functions between positive and negative CG beads in the MSPEs with $n_{arm} = 8$ (A), $n_{arm} = 16$ (B) and $n_{arm} = 40$ (C), respectively.

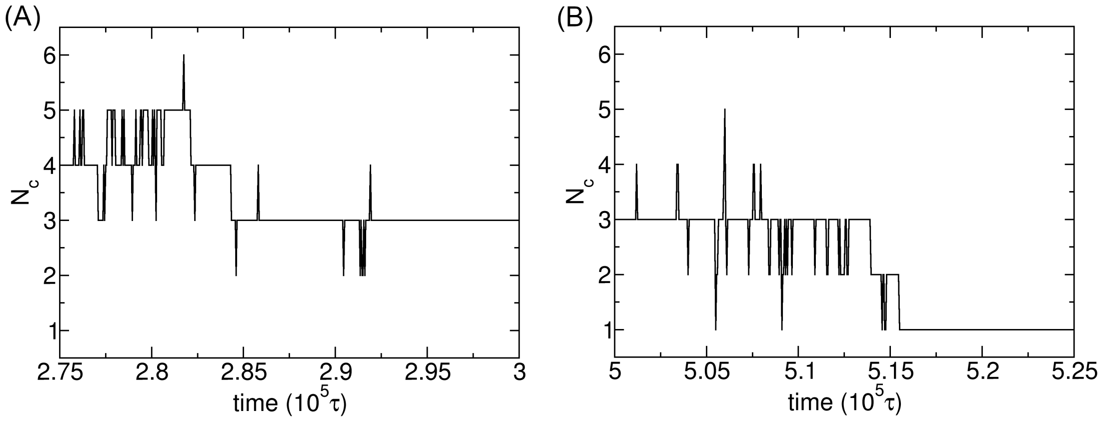

Figure S4: Evolution of cluster numbers N_c during the time periods when the clustering occurs in the system including MSPEs with $n_{arm} = 16$ under $\lambda_{\mathrm{D}} = 2\sigma$. (A) corresponds to the time from $2.75 \times 10^5\tau$ to $3.0 \times 10^5\tau$, (B) corresponds to the time from $5.0 \times 10^5\tau$ to $5.25 \times 10^5\tau$.

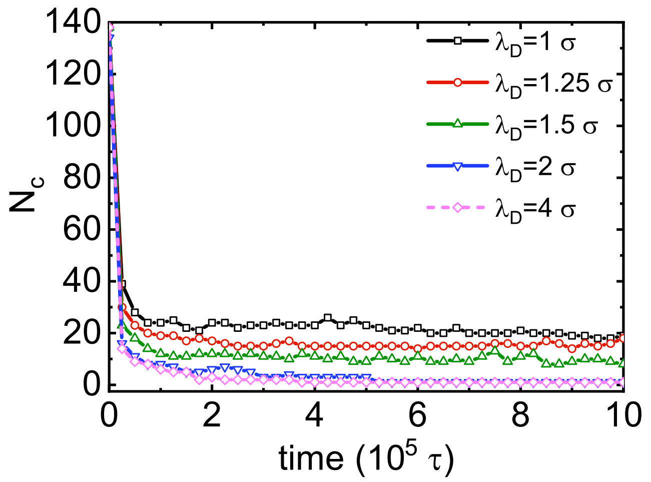

Figure S5: Time evolution of number of clusters formed by MSPEs with $n_{arm} = 16$ under different ionic strengths.

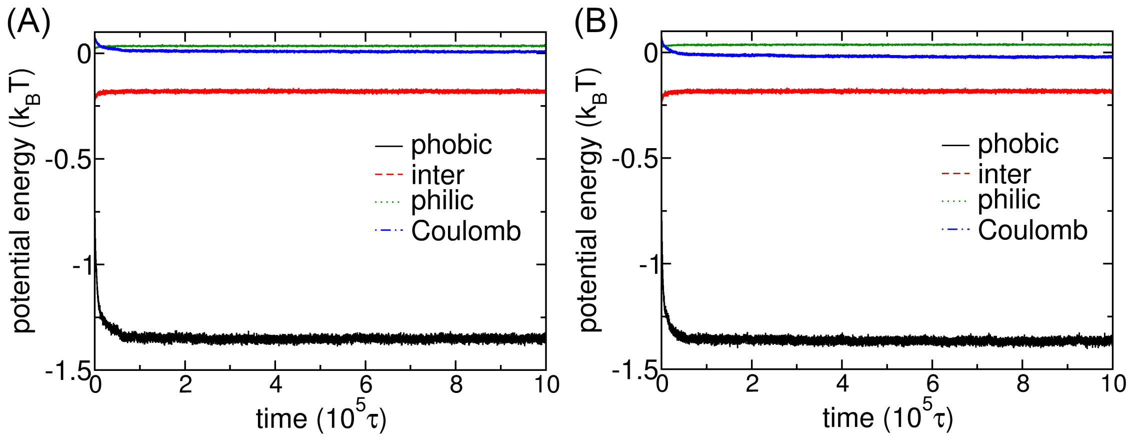

Figure S6: Time evolution of Lennard-Jones and Coulomb potential energies between different components, including the hydrophobic interactions (phobic), the interactions between hydrophilic and hydrophobic arms (inter), the steric repulsion (philic), and Coulomb interactions (Coulomb). (A) is the results of the MSPEs with $n_{arm} = 16$ under $\lambda_D = 1.5\ \sigma$, (B) is under $\lambda_D = 2\ \sigma$.

Validation of stability of self-assembly structures

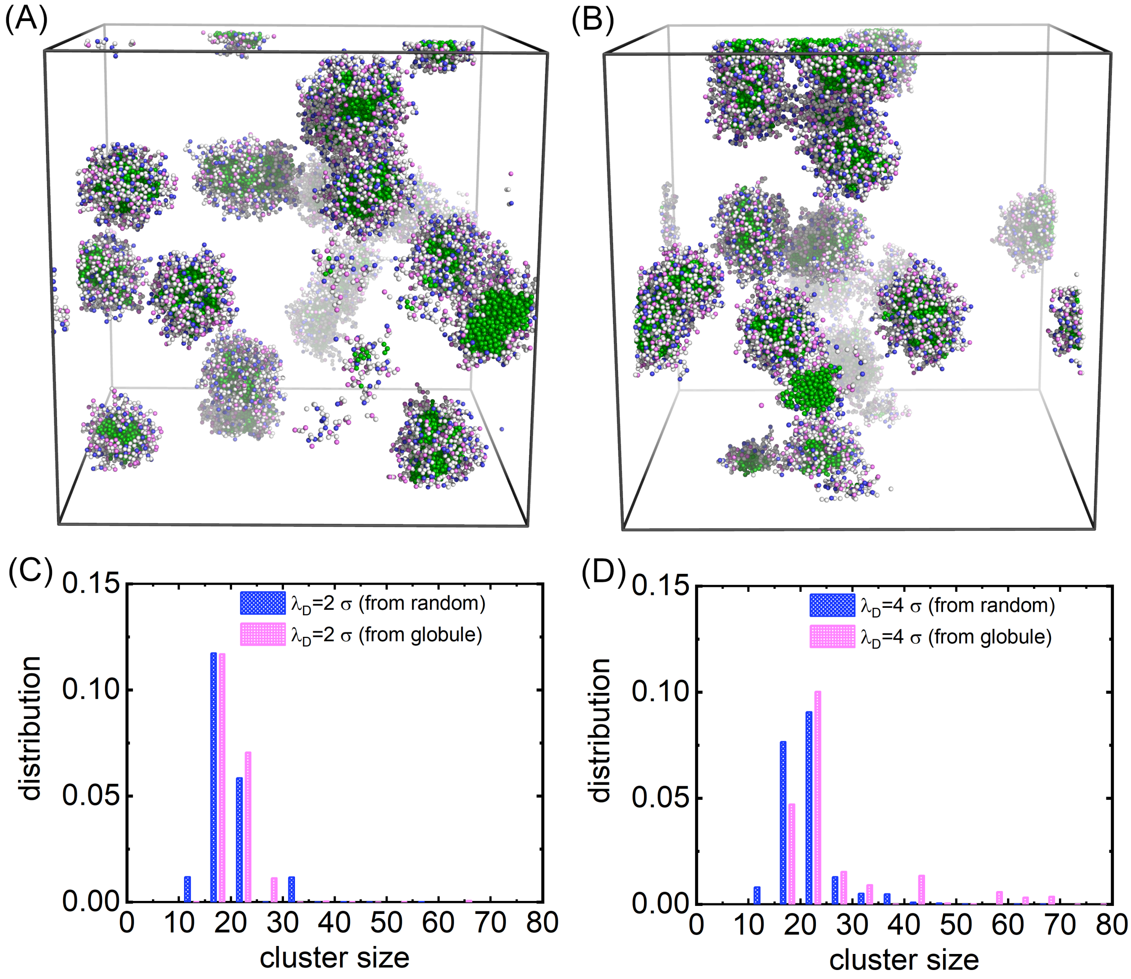

Figure S7: Self-assembly structures formed by MSPEs with n_{arm}=8 at (A) $\lambda_{\rm D}$=2.0σ and (B) $\lambda_{\rm D}$=4.0σ, from the globular initial configuration. The comparisons of normalized cluster size distribution for micelles with randomly distributed and globular initial configuration are shown in (C) $\lambda_{\rm D}$=2.0σ and (D) $\lambda_{\rm D}$=4.0σ.

We also simulated the systems starting from an energy minimised full globular state with diameter $d = 40\sigma$, and we found that the spherical, wormlike micelles are also observed in the systems. Figure S7 shows the comparisons of self-assembly structures formed by MSPEs with the number of arms n_{arm}=8, from randomly distributed initial configurations (in the manuscript) and globular initial state. The overall self-assembly morphologies do not depend on the initial configuration obviously, the spherical micelles

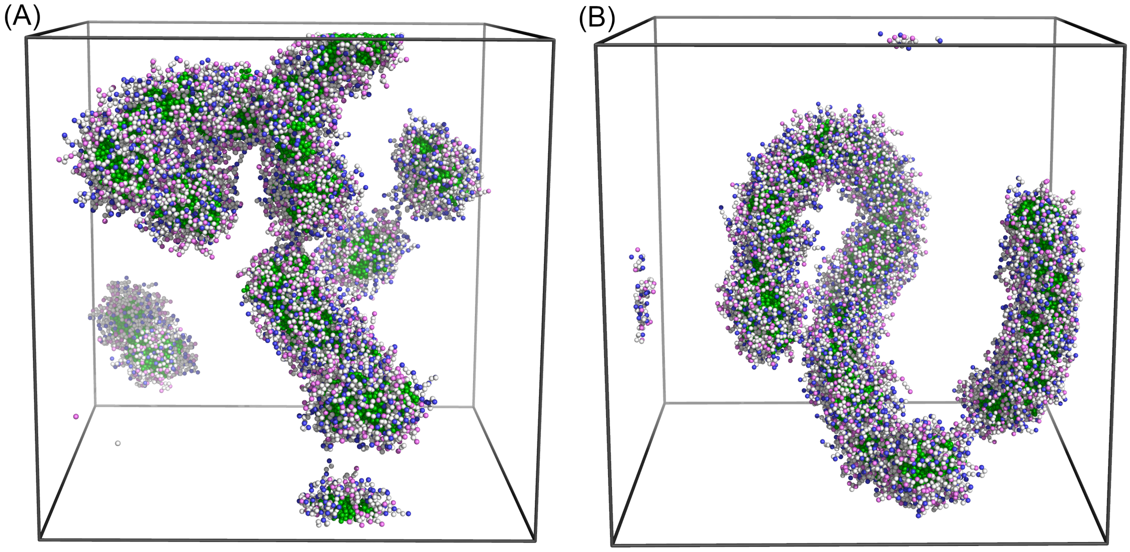

Figure S8: Self-assembly structures formed by MSPEs with n_{arm}=16 at (A) λ_D=1.5σ and (B) λ_D=2σ, from the globular initial configuration.

are also observed at λ_D=2.0σ, and the micelles with inter-connection are formed at λ_D=4.0σ, from the snapshots shown in Figure S7A and B. We also calculated the cluster size distribution for the two systems with globular initial configuration, and the results are also similar as those shown in the manuscript (Figure S7C and D).

In addition, we also checked the probability of wormlike micelle formation from globular initial state, and the representative self-assembly structures are shown in Figure S8, which are formed by the MSPEs with n_{arm}=16 at λ_D=1.5σ and λ_D=2σ. The self-assembly structures are similar as Figure 3 and 6 in the manuscript. So we believe that the self-assembly structures like spherical or wormlike micelles are the results after the systems reach thermodynamical equilibrium state, and the results are independent on the initial configurations.
